# Hedgehog/GLI Signaling Activates Suppressor of Cytokine Signaling 1 (*SOCS1*) in Epidermal and Neural Tumor Cells

**DOI:** 10.1371/journal.pone.0075317

**Published:** 2013-09-10

**Authors:** Sandra Laner-Plamberger, Florian Wolff, Alexandra Kaser-Eichberger, Stefan Swierczynski, Cornelia Hauser-Kronberger, Anna-Maria Frischauf, Thomas Eichberger

**Affiliations:** 1 Department of Molecular Biology, University of Salzburg, Salzburg, Austria; 2 Department of Blood Group Serology and Transfusion Medicine, University Hospital of Salzburg, Paracelsus Medical University, Salzburg, Austria; 3 Spinal Cord Injury & Tissue Regeneration Center Salzburg (SCI-TReCS), Paracelsus Medical University (PMU), Salzburg, Austria; 4 Department of Ophthalmology, University Hospital, Salzburg, Paracelsus Medical University, Salzburg, Austria; 5 Department of Pathology, University Hospital of Salzburg, Paracelsus Medical University, Salzburg, Austria; Indiana University School of Medicine, United States of America

## Abstract

Sustained hedgehog (Hh) signaling mediated by the GLI transcription factors is implicated in many types of cancer. Identification of Hh/GLI target genes modulating the activity of other pathways involved in tumor development promise to open new ways for better understanding of tumor development and maintenance. Here we show that SOCS1 is a direct target of Hh/GLI signaling in human keratinocytes and medulloblastoma cells. SOCS1 is a potent inhibitor of interferon gamma (IFN-y)/STAT1 signaling. IFN-у/STAT1 signaling can induce cell cycle arrest, apoptosis and anti-tumor immunity. The transcription factors GLI1 and GLI2 activate the SOCS1 promoter, which contains five putative GLI binding sites, and GLI2 binding to the promoter was shown by chromatin immunoprecipitation. Consistent with a role of GLI in SOCS1 regulation, STAT1 phosphorylation is reduced in cells with active Hh/GLI signaling and IFN-у/STAT1 target gene activation is decreased. Furthermore, IFN-у signaling is restored by shRNA mediated knock down of SOCS1. Here, we identify SOCS1 as a novel Hh/GLI target gene, indicating a negative role of Hh/GLI pathway in IFN-y/STAT1 signaling.

## Introduction

Hh/GLI signaling is of central importance during vertebrate embryonic development and also plays a crucial role in regulating cell proliferation and differentiation in the adult organism. A rapidly growing number of facts has linked aberrant Hh pathway activity to tumorigenesis. It has been shown that malignant transformations in organs like skin, brain, prostate, the lung and many more are involving irregular Hh signaling (reviewed in [1,2]).

Hh signaling is canonically activated by binding of the signaling molecule Hh to its transmembrane receptor patched (PTCH), abrogating the inhibitory effect of PTCH on the signal transducer smoothened (SMO). Activation of SMO leads to the stabilization of the activator form of GLI transcription factors. Active GLI proteins then translocate from the primary cilium, where pathway activation takes place, to the nucleus to drive Hh target gene transcription (reviewed in [3–5]).

First indications for a tumor promoting function of Hh pathway activity was found in patients suffering from the autosomal dominant hereditary disease BCNS (Gorlin Syndrome) characterized by multiple Basal Cell Carcinomas (BCCs) and rare cases of medulloblastoma (MB) and rhabdomyosarcoma (RMS). The molecular basis of this phenotype, but also for spontaneously developed BCCs and MBs not associated with Gorlin syndrome, is most frequently the mutational inactivation of the pathway repressor PTCH [6–8]. Further causes for spontaneous BCCs and MBs can be activating mutations in SMO [9] or loss of function mutations in SUFU [8,10]. The importance of the hedgehog pathway in BCC, MB and RMS development has been further demonstrated by numerous transgenic and knock out mouse models [11–13]. Recently Hh signaling has been shown to interact with several other signaling pathways like EGF, TGF-β, WNT, NOTCH and IFN-y, which are playing key roles in different cellular processes, but also strongly influence tumor growth and metastasis [14–21]. Characterizing such interactions is an important aim in developing new therapeutic strategies for cancer treatment.

Suppressor of cytokine signaling 1 (SOCS1) is a member of a protein family mainly known as negative regulators of cytokine induced JAK-STAT signal transduction (reviewed in [22–24]). The SOCS family consists of eight members, SOCS1 to 7 and the cytokine inducible SH2 containing protein CIS. Characteristic for all SOCS family members are a central SH2 domain and a highly conserved C-terminal SOCS box motive. SOCS1 contains an additional N-terminal kinase inhibitory domain (KIR). The SH2 domain and the KIR motive are both required for efficient binding to activated JAK kinases and subsequent blocking of signaling by preventing STAT phosphorylation [25–27].

In mouse models, SOCS1 was shown to specifically antagonize STAT1 and its functions downstream of IFN-у. SOCS1 knockout mice die within the first weeks after birth because of hyper-responsiveness to IFN-у resulting from increased STAT1 phosphorylation and IFN-у/STAT1 target gene expression. They can be rescued by concurrent IFN-y knock out [28,29]. Accordingly overexpression of SOCS1 in transgenic animals or in cultured cells cause strongly reduced IFN-у responsiveness [30–35].

The roles of SOCS1 in tumorigenesis are diverse and strongly depend on the origin or type of the tumor. SOCS1 may either promote or suppress tumorigenesis: Tumor suppressive activity of SOCS1 was observed in SOCS1-/- knockout mice, which develop colitis-induced colon tumors [36]. Deletion or silencing of SOCS1 in human hepatocellular carcinoma (HCC) [37], acute myeloid leukemia [38] and gastric cancer [39] also points to the anti-tumor potential of SOCS1. In contrast, SOCS1 acts as an oncogene by inhibiting the IFN-у mediated effects on cancer cells such as enhanced anti-tumor immunity, cell cycle arrest, apoptosis and reduced angiogenesis. Depletion of SOCS1 negatively affects various tumor types like melanoma and neuroendocrine tumors [40,41] supporting an oncogenic potential for the STAT1 inhibitor SOCS1.

Here we show that SOCS1 is a direct target of Hh/GLI signaling in human keratinocytes and a medulloblastoma cell line. STAT1 phosphorylation and IFN-y target gene activation are downregulated upon expression of GLI transcription factors. This effect can be reversed by shRNA mediated knockdown of SOCS1, suggesting that Hh signaling in tumor cells blocks IFN-у mediated anti-tumor effects via activation of SOCS1.

## Results

### SOCS1 expression is enhanced in response to Hh/GLI signaling

**Figure 1:**
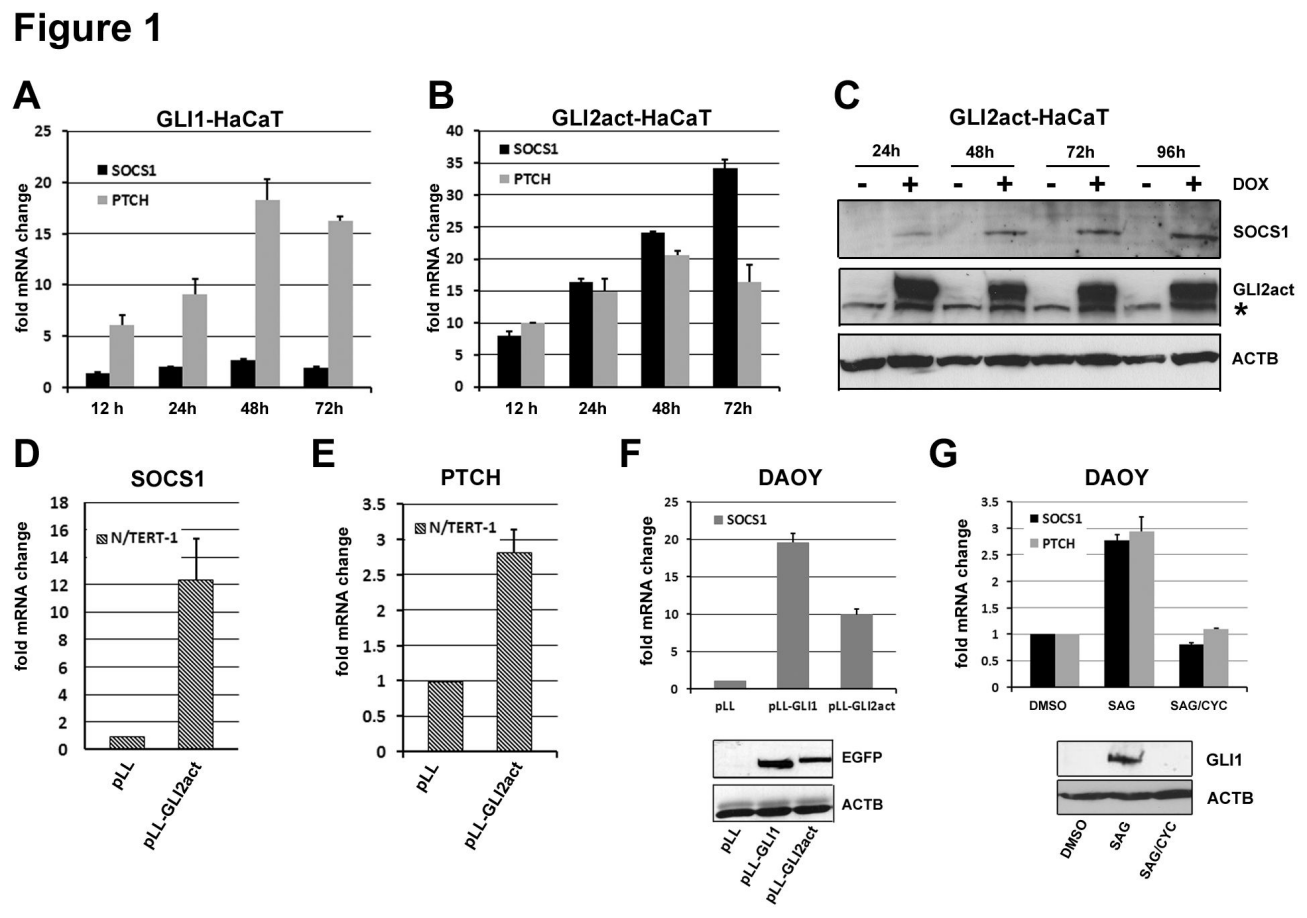
Hh/GLI signaling induces SOCS1 expression. **A**) and **B**) qRT–PCR of SOCS1 (black bars) and PTCH (grey bars) mRNA levels in HaCaT keratinocytes expressing GLI1 (GLI1-HaCaT) (A) or GLI2act (GLI2act-HaCaT) (B) under doxycycline (DOX) control for the times indicated. **C**) Western blot of SOCS1 and GLI2act protein level in DOX treated and untreated GLI2act-HaCaT cells. Beta-actin (ACTB) was used as loading control. **D**) and **E**) qRT–PCR of SOCS1 and PTCH expression in the keratinocyte cell line N/TERT-1 retrovirally transduced with GLI2act (pLL-GLI2act) or enhanced green fluorescent protein (EGFP) (pLL) as control. Cells were assayed 48h post infection. Fold change refers to mRNA ratio of GLI2act to EGFP expressing cells. **F**) qRT-PCR of SOCS1 mRNA in DAOY cells retrovirally transduced with EGFP tagged GLI1 (pLL-GLI1), GLI2act (pLL-GLI2act) or EGFP (pLL). Lower panel: Western blot of GLI1 and GLI2act transgene expression using EGFP antibody. **G**) DAOY cells were treated with Hh pathway agonist SAG alone or in combination with the antagonist cyclopamine (CYC) for 120h and analyzed for the expression of SOCS1 and PTCH by qRT-PCR. Controls were treated with DMSO only. Lower panel: Activation of the Hh pathway was monitored by Western blot using a GLI1 specific antibody. Error bars represent ± SD of biological triplicates. * unspecific signal.

SOCS1, a member of the SOCS protein family, was identified as a GLI target gene in HaCaT keratinocyte cell lines inducibly expressing either GLI1 (GLI1-HaCaT) or GLI2 activator form (GLI2act-HaCaT) [42]. Other members of the SOCS family showed no response to either GLI transcription factor. qRT-PCR demonstrated strong upregulation of SOCS1 mRNA in response to GLI2act (up to 35 fold) and moderate activation due to GLI1 overexpression (Figure 1A and B). A comparable increase of SOCS1 protein in response to GLI2act expression was seen by Western blot (Figure 1C). Retrovirally transduced GLI2act in the keratinocyte cell line N/TERT-1 can also induce SOCS1 mRNA expression (Figure 1D). The canonical Hh/GLI target PTCH was used as positive control for GLI activity (Figure 1A, B and E). While in HaCaT cells induction of SOCS1 by GLI1 is moderate compared to GLI2act (Figure 1A and B), strong expression of SOCS1 was found in the medulloblastoma cell line DAOY in response to either GLI1 or GLI2act expression (Figure 1F). To further support that GLI mediated induction of SOCS1 is due to a physiological amount of Hh signaling rather than excessive overexpression we activated Hh signaling in DAOY cells with the smoothened agonist SAG [43]. As expected, SAG treatment of DAOY cells leads to a significant increase in PTCH expression (Figure 1G, grey bars) and robust expression of endogenous GLI1 protein (Figure 1G, lower panel). SOCS1 expression induced by SAG was comparable to PTCH and was completely ablated by the pathway antagonist cyclopamine (CYC) (Figure 1G, black bars).

**Figure 2:**
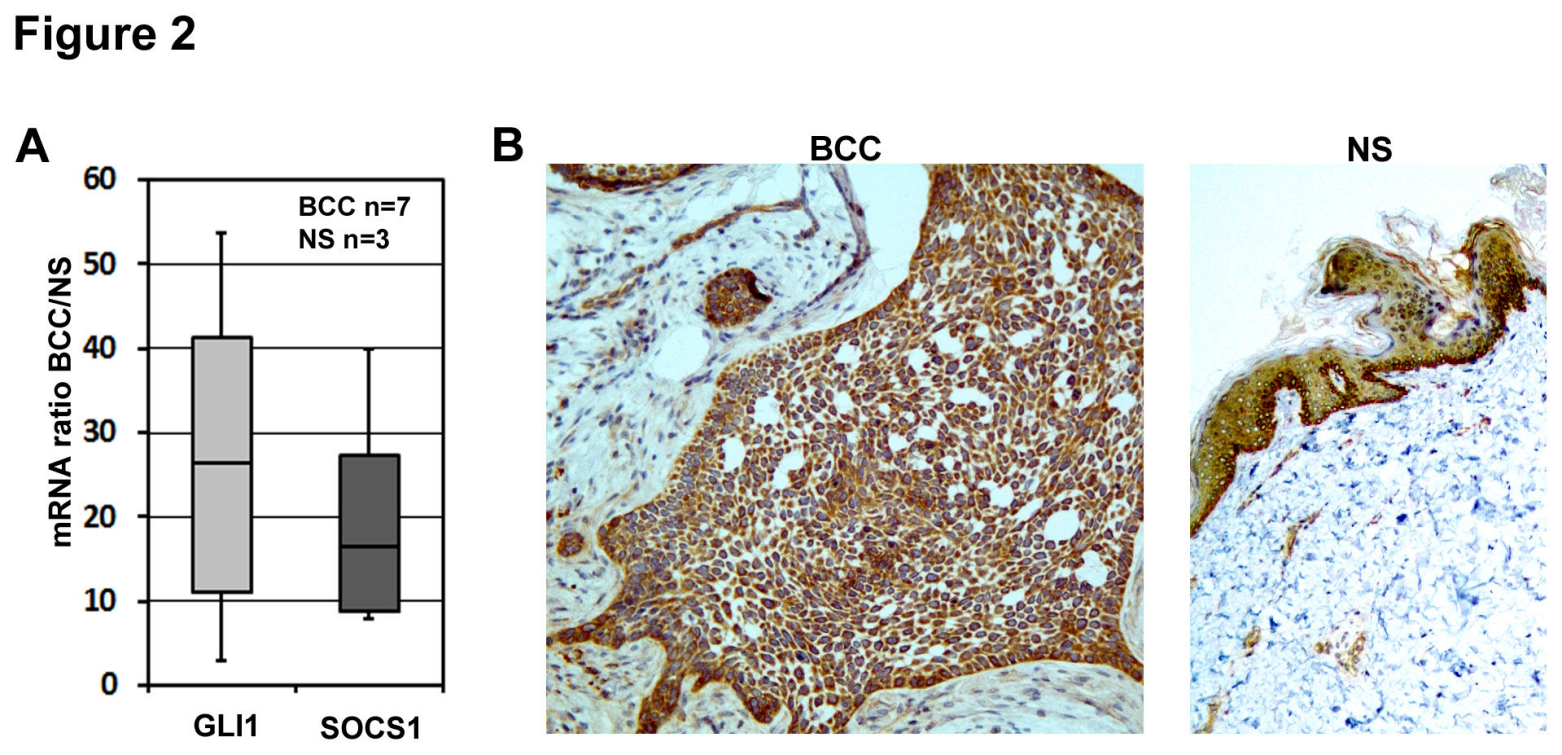
SOCS1 is expressed in basal cell carcinoma (BCC). **A**) mRNA levels of hedgehog target genes GLI1 and SOCS1 in biopsies of human BCCs (n=7) and normal human skin samples (NS) (n=3) by qRT-PCR. Data were normalized to RPLP0 and represent mean values of all tested BCC and NS samples. Fold change refers to the ratio of BCC to NS. **B**) Immunostaining of sections of human BCCs (left) and normal skin (NS, right) with SOCS1 antibody.

BCC is a keratinocyte derived tumor characterized by constitutive activation of the Hh pathway [6–13]. We therefore analyzed samples of human BCCs to determine whether and at what level SOCS1 is expressed in these tumors. Seven BCC samples showing characteristic high expression of GLI1 [7,44,45] were found to also strongly express SOCS1 mRNA compared to three normal human skin biopsies (Figure 2A). Immunohistochemical staining of human BCC paraffin sections showed strong and specific staining of SOCS1 protein throughout the tumor islands and in some infiltrated areas of the surrounding stroma thus supporting qRT-PCR data (Figure 2B, left). Furthermore, staining of sections of paraffin-embedded human normal skin showed significant expression of SOCS1 protein in basal keratinocytes, which are sites of GLI expression [46]. This agrees with previous studies of SOCS1 expression in skin [47] (Figure 2B, right).

### SOCS1 is a direct transcriptional target of the GLI transcription factors

**Figure 3:**
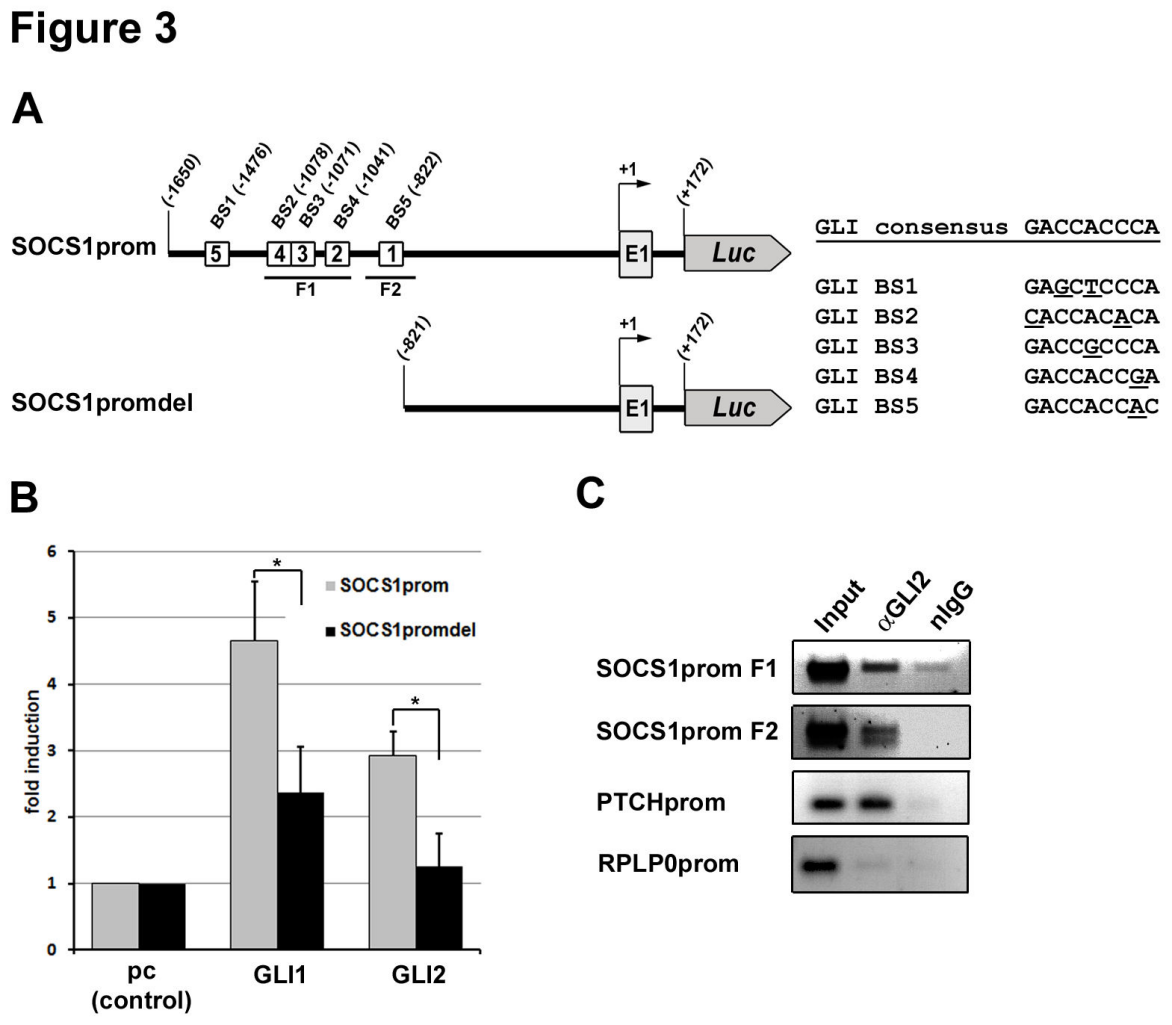
SOCS1 is a direct transcriptional target of GLI. **A**) Graphical overview of the cloned SOCS1 promoter region containing 5 putative GLI binding sites. Numbers refer to the transcription start site (RefSeq NM_003745.1). Sequences of putative GLI binding sites are listed on the right. Bases differing from GLI consensus sequence are underlined. **B**) Luciferase assay of a 1822bp fragment of the human SOCS1 promoter (SOCS1prom, see A) and deletion construct SOCS1promdel (see A). HaCaT cells were co-transfected with SOCS1 reporter and GLI expression plasmids as indicated. +/-SD refers to quadruplicate samples. **C**) Chromatin immunoprecipitation shows specific binding of GLI2 to the SOCS1 promoter. Chromatin isolated from doxycycline (DOX) treated GLI2act-HaCaT cells was precipitated with either GLI2 specific antibody (αGLI2) or unspecific (normal IgG) antibody as control. Two fragments (F1 and F2) spanning BS2, BS3, and BS4 or BS5 were amplified from the SOCS1 promoter by PCR. As positive control a 148-bp fragment (PTCHprom) from the PTCH promoter was used [49] and a 284-bp fragment (RPLP0prom) from the human RPLP0 promoter served as negative control [70]. * P < 0.001.

To find out whether SOCS1 expression is directly regulated by the GLI transcription factors, we searched for putative GLI binding sites upstream of the transcriptional start site of SOCS1. Using ScanACE [48] and a search motif based on Winklmayr et al. [49] we identified a cluster of five sites within a 600bp region, located 822bp upstream of the transcriptional start site (Figure 3A). All of these potential binding sites differ in at least one position from the GLI consensus sequence [50] (Figure 3A, right) and were previously shown to be active in luciferase assays [49]. To confirm the role of the GLI binding sites in SOCS1 upregulation, we cloned a 1478bp fragment (-1650 to +172) of the human SOCS1 promoter containing the transcriptional start site and the first exon into a luciferase reporter plasmid (SOCS1prom) and a control promoter with all five GLI binding sites deleted (SOCS1promdel) (Figure 3A). As expected, luciferase expression from the SOCS1 reporter was significantly activated by both GLI1 and GLI2 (Figure 3B). Deletion of the GLI binding site cluster led to strongly reduced luciferase activity (Figure 3B), supporting direct regulation of SOCS1 by the transcription factors GLI1 and GLI2. To show physical interaction of the GLI proteins with this promoter region *in vivo*, chromatin immunoprecipitation was done in GLI2act-HaCaT cells, demonstrating that GLI2 binds to the SOCS1 promoter fragments F1 and F2 (Figure 3A), which contain putative GLI binding sites (Figure 3C). Together these results show that SOCS1 is a direct target of the GLI transcription factors.

### Diminished IFN-у/STAT1 signaling in cells with activated Hh/GLI pathway

**Figure 4:**
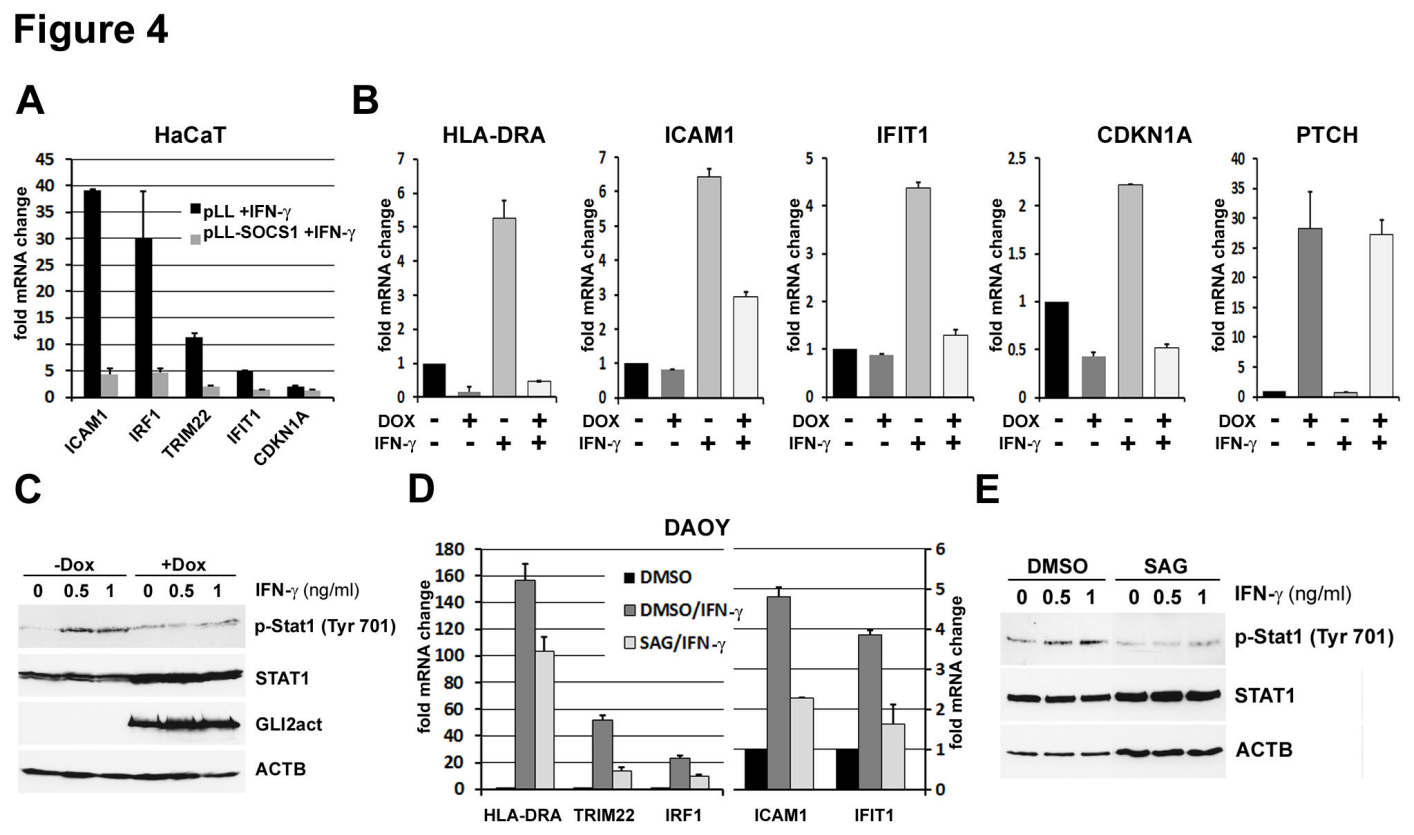
IFN-у /STAT1 signaling is downregulated in cells with active Hh/GLI signaling. **A**) The effect of SOCS1 expression on IFN-у/STAT1 signaling was analyzed in HaCaT cells expressing FLAG tagged SOCS1 (pLL-SOCS1) or empty viral vector as control (pLL). 48h hour post transfection cell were treated with 1 ng/ml IFN-у for 6h and assayed for mRNA levels of known IFN-у target genes by qRT-PCR. Fold change refers to the ratio of IFN-у treated to untreated samples. **B**) qRT-PCR of GLI2act-HaCaT cells expressing GLI2 (+DOX) for 72h and subsequently treated with 1 ng/ml IFN-у for 6h. mRNA levels IFN-у target genes (HLA-DRA, ICAM1, IFIT1, and p21) are shown as ratios to untreated control. PTCH was used as marker for GLI2 activity. **C**) Western blot showing STAT1 phosphorylation in GLI2act-HaCaT cells treated with doxycycline (DOX) for 72h followed by a 2h treatment with IFN-у. **D**) qRT-PCR of IFN-у target gene expression in DAOY cells treated with SAG or DMSO control for 144h followed by 4h of IFN-у treatment. mRNA levels are shown as ratio of treated to untreated samples. Data are given as mean ± SD of biological triplicates. **E**) STAT1 phosphorylation in SAG/IFN-у treated DAOY was analyzed by Western blot.

Having shown that SOCS1 is a direct GLI target, we asked whether GLI expression affects IFN-у signaling via modulation of SOCS1 and consequently STAT1, the main signal transducer of type II interferon signaling [51,52]. Federici et al. have shown that SOCS1 overexpression in HaCaT keratinocytes inhibits the activation of STAT1 and thus impairs IFN-y dependent target gene expression [33]. Results of qRT-PCR analysis of IFN-y target genes in HaCaT cells overexpressing SOCS1 are in agreement with previously published data [33] (Figure 4A). We then treated HaCaT cells expressing SOCS1 (pLL-SOCS1) with IFN-у and quantified expression of the known IFN-у target genes ICAM1, IRF1, TRIM22, IFIT1 and the cell cycle inhibitor p21 (CDKN1A) (reviewed in [51]) by qRT-PCR (Figure 4A). As expected, cells transduced with EGFP as control (Figure 4A, black bars) showed a strong response to IFN-у as evident from the dramatic increase in ICAM1 (37 fold over IFN-у untreated), IRF1 (30 fold), TRIM22 (12 fold), IFIT1 (5 fold) and CDKN1A (3 fold), while in cells expressing SOCS1 (pLL-SOCS1) very little or no in IFN-у target gene activation was observed (Figure 4A, grey bars).

We then asked whether the effect of SOCS1 on IFN-y target gene expression could be triggered by GLI expression. GLI2 expression in the inducible GLI2act-HaCaT cells led to a dramatically reduced IFN-у response almost completely abolishing activation of ICAM1, HLA-DRA, IFIT1 and CDKN1A (Figure 4B), while expression of the canonical Hh target gene PTCH was not affected by IFN-у, but increased by GLI as expected [53,54]. In agreement with reduced IFN-у signaling in GLI2act expressing HaCaT cells Western blot demonstrated a significant reduction in STAT1 phosphorylation (Figure 4C). To extend the result to the context of a different cell type and avoiding overexpression of GLI, we tested the IFN-у response in DAOY cells treated with the hedgehog pathway agonist SAG. As observed for HaCaTs, DAOY cells showed a strong transcriptional response to IFN-у. In SAG treated cells both IFN-у target gene expression and phosphorylation of STAT1 were significantly reduced compared to DMSO treated controls (Figure 4D and E). In summary, these results indicate that Hh pathway activation impairs IFN-у/STAT1 signaling.

### Knock down of SOCS1 restores IFN-у target gene activation in the presence of Hh/GLI signaling

**Figure 5:**
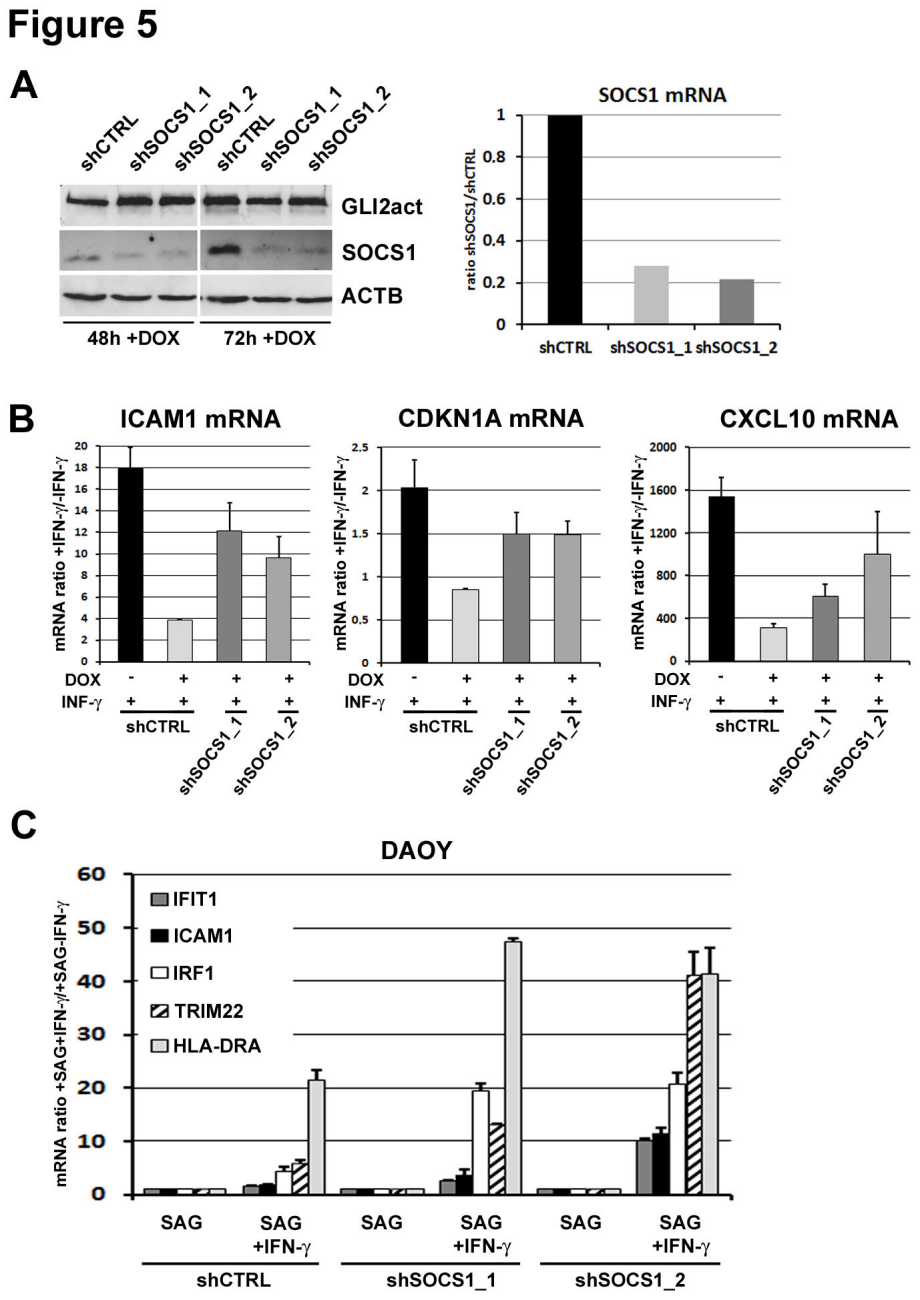
SOCS1 knock down restores IFN-у/STAT1 signaling in cells with activated Hh signaling. **A**) Western blot analysis of SOCS1, GLI2, and ACTB in GLI2act-HaCaT cells transduced with two shRNAs directed against human SOCS1 (shSOCS1_1, shSOCS1_2) and control shRNA (shCTRL) expressing GLI2 for the time indicated (left panel). SOCS1 mRNA levels were also analyzed by qRT-PCR in cells expressing GLI2 for 48h. **B**) qRT-PCR of IFN-у target genes (CXCL10, CDKN1A, and ICAM1) measured in GLI2act-HaCaT cells after expressing GLI2 for 48h (+ DOX) with subsequent exposure to 1ng/ml recombinant IFN-у for 6h. **C**) qRT-PCR of IFN-у target gene activation (HLA-DRA, ICAM1, IFIT1, TRIM22 and IRF1) in DAOY cell lines stably expressing either shSOCS1_1 and shSOCS1_2 or unspecific control shRNA (shCTRL). Cells were pretreated with 200 nM SAG for 120h to activate the Hh pathway and subsequently incubated with 1ng/ml recombinant IFN-у for 6h. mRNA levels are shown as ratio of IFN-у treated to untreated samples. Data are given as mean ± SD of biological triplicates.

To test whether SOCS1 is directly responsible for the repression of IFN-у/STAT1 signal transduction in the presence of Hh signaling, we used an shRNA approach to knock down SOCS1. GLI2act-HaCaT cells were transduced with shSOCS1_1, shSOCS1_2 or control shRNA. The efficiency of the knock down was evaluated by Western blot and qRT-PCR (Figure 5A). Next, we analyzed mRNA levels of selected IFN-у targets in GLI2act-HaCaT cells stably expressing shSOCS1_1, shSOCS1_2 or shCTRL. As predicted, knock down of SOCS1 in cells with active hedgehog signaling partly restores IFN-у target gene activation: HaCaT cells expressing GLI2act and SOCS1 shRNA showed significantly increased activation of the IFN-у target genes CXCL10, CDKN1A and ICAM1 compared to cell lines expressing GLI2 and control shRNA (Figure 5B). Similar results were obtained in SAG treated DAOY cells transduced with shSOCS1_1 or shSOCS1_2. Again, knock down of SOCS1 strongly enhances IFN-у target gene activation in SAG treated cells (Figure 5C).

### Reduced anchorage-independent growth of DAOY cells in response to SOCS1 knock down

**Figure 6:**
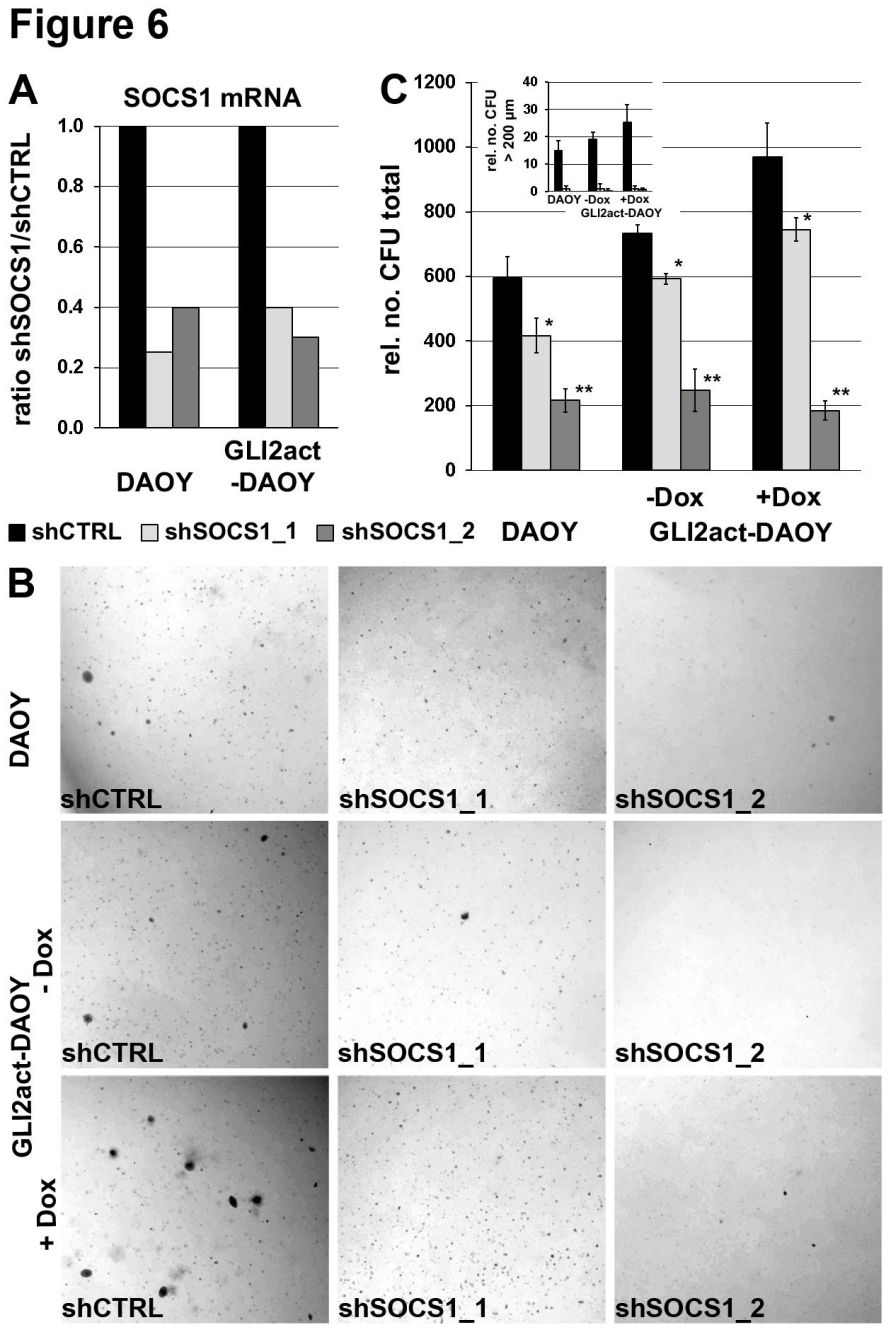
Reduced anchorage-independent growth of Hh-active DAOY cells in response to SOCS1 knock down. **A**) SOCS1 mRNA levels were analyzed by qRT-PCR in DAOY and GLI2act-DAOY (24h GLI2act expression), showing efficient SOCS1 knock down. Black bar: shCTRL, light grey bar: shSOCS1_1, dark grey bar: shSOCS1_2. **B**) Anchorage independent growth of DAOY cells (top) or GLI2act-DAOY (middle: uninduced GLI2act DAOY (-Dox), bottom: induced GLI2act DAOY (+Dox)) expressing shCTRL, shSOCS1_1 or shSOCS1_2. Number and size of colonies are enhanced in response to GLI2act expression and reduced in presence of SOCS1 knock down. **C**) Quantification of assays shown in B). The large diagram shows the total number of all colonies, the small diagram refers to the number of colonies with a diameter larger than 200 µm. rel. no. CFU relative number of colony forming units. Black bar: shCTRL, light grey bar: shSOCS1_1, dark grey bar: shSOCS1_2. Error bars represent ± SD of biological quadruplicates, * P < 0.05, ** P < 0.01.

Untreated DAOY cells display a sustained, low level of Hh pathway activity (reviewed in [55]) and are known to form colonies in colony formation assays [56–58]. Having shown that high levels of Hh signaling impairs IFN-y signaling by SOCS1 activation, we explored the influence of SOCS1 on Hh driven tumor growth. An inducible GLI2act expressing DAOY cell line (GLI2act-DAOY) and unmodified DAOY cells were subjected to retrovirally induced SOCS1 knock down. The efficiency of shRNA mediated knock down was evaluated by qRT-PCR (Figure 6A). As expected, untreated DAOY cells and uninduced GLI2act-DAOY cells (-Dox) when transduced with control shRNA form a small number of colonies, which can be further reduced by SOCS1 shRNA (Figure 6B upper and middle row). Induced expression of GLI2act (GLI2act-DAOY +Dox) leads to a higher number of larger colonies (> 200 µm) (Figure 6B bottom, shCTRL). SOCS1 knock down strongly reduces the number of colonies (Figure 6B, shSOCS1_1, shSOCS1_2 vs shCTRL) and colonies larger than 200 µm are completely absent (Figure 6B and 6C, small diagram). These results demonstrate that colony formation of DAOY cells is strongly enhanced in the presence of GLI2act. Furthermore, a knock down of SOCS1 antagonizes colony formation, leading to a significant reduction of colony number and size (Figure 6C). These results indicate that upregulated expression of SOCS1 may contribute to tumor growth.

## Discussion

The oncogenic potential of uncontrolled activation of Hh signaling has been demonstrated extensively in the past years. Interaction of Hh signaling with a number of other pathways can enhance tumorigenesis and tumor growth. Here we describe an inhibitory interaction of Hh/GLI signaling with the IFN-у/STAT1 pathway. IFN-у/STAT1 signaling can have tumor suppressor function and IFN-у treatment is recommended and tested in tumor therapy [59]. Mice insensitive to IFN-у (STAT-/-, IFN-уR-/-) exhibit enhanced chemically induced tumor development and are more susceptible to transplanted tumors [60,61]. Moreover, 33% of all tested melanoma and lung adenocarcinoma cell lines have inactivating mutations in at least one IFN-у pathway component [60], suggesting that inactivation of the IFN-у pathway can lead to evasion of cytokine induced cell cycle arrest and cytokine mediated tumor surveillance.

SOCS1, a specific inhibitor of IFN-y/STAT1 signaling, is activated by IFN-y/STAT1 thus creating a negative feedback leading to a block of STAT1 phosphorylation (reviewed in 26,62). Here we show that SOCS1 is a direct transcriptional target of the oncogenic Hh signal mediators GLI1 and GLI2. HaCaT keratinocytes and DAOY cells with elevated Hh signaling due to either GLI1 or GLI2 overexpression or treatment with the Hh pathway agonist SAG show highly elevated levels of SOCS1. This results in a significantly decreased expression of IFN-у target genes such as IRF1, ICAM1, CDKN1A and HLA-DRA. The data by Umeda et al. showing a protective function of Hh expression on IFN-y induced cytotoxicity in pancreatic beta cells [63] may result from a similar interaction, though a contribution of SOCS1 was not investigated in this study.

A knock out of STAT1 leads to complete loss of IFN-y responsiveness [64] and mice deficient for SOCS1, a STAT1 inhibitor, die from IFN-у mediated, dramatically increased systemic inflammation [28,29]. Here we show that the levels of active, phosphorylated STAT1 are reduced in response to activation of the Hh pathway. Furthermore, we demonstrate that a knock down of SOCS1 in cells with activated Hh pathway restores IFN-y driven target gene activation. These data support the hypothesis that hedgehog driven SOCS1 expression leads to reduced levels of phosphorylated STAT-1 and IFN-у susceptibility.

Upregulation of SOCS1 in response to Hh signaling is observed not only in HaCaT keratinocytes and DAOY medulloblastoma cells, but also in samples of human BCC. Compared to normal epidermal keratinocytes, BCCs are characterized by lower expression of IFN-y target genes ICAM1 and HLA-DRA [65,66]. This may be due to elevated SOCS1 expression and is in agreement with reduction of HLA-DRA and ICAM-1 expression seen in GLI expressing keratinocytes (Figure 4B).

High levels of SOCS1 have been reported in breast cancer and some melanomas [47,67–69]. However, also silencing of SOCS1 has been described in various tumour types [37–41]. To further define the role of SOCS1 on tumorigenesis in the context of Hh signaling, we used DAOY cells in a colony formation assay. We observed a reduction in the number and size of colonies in the presence of shRNA directed against SOCS1 compared to control shRNA, indicating that high levels of SOCS1 expression promote tumor growth. The observed reduction of colonies even in the absence of exogenous IFN-y is probably resulting from a basal level of endogenous IFN-y signaling, also shown by the presence of IFN-y transcript (see also Figure S1) and a low, but detectable amount of phosphorylated STAT-1 in DAOY cells, which is decreased by induction of Hh signaling through SAG (Figure 4E).

**Figure 7:**
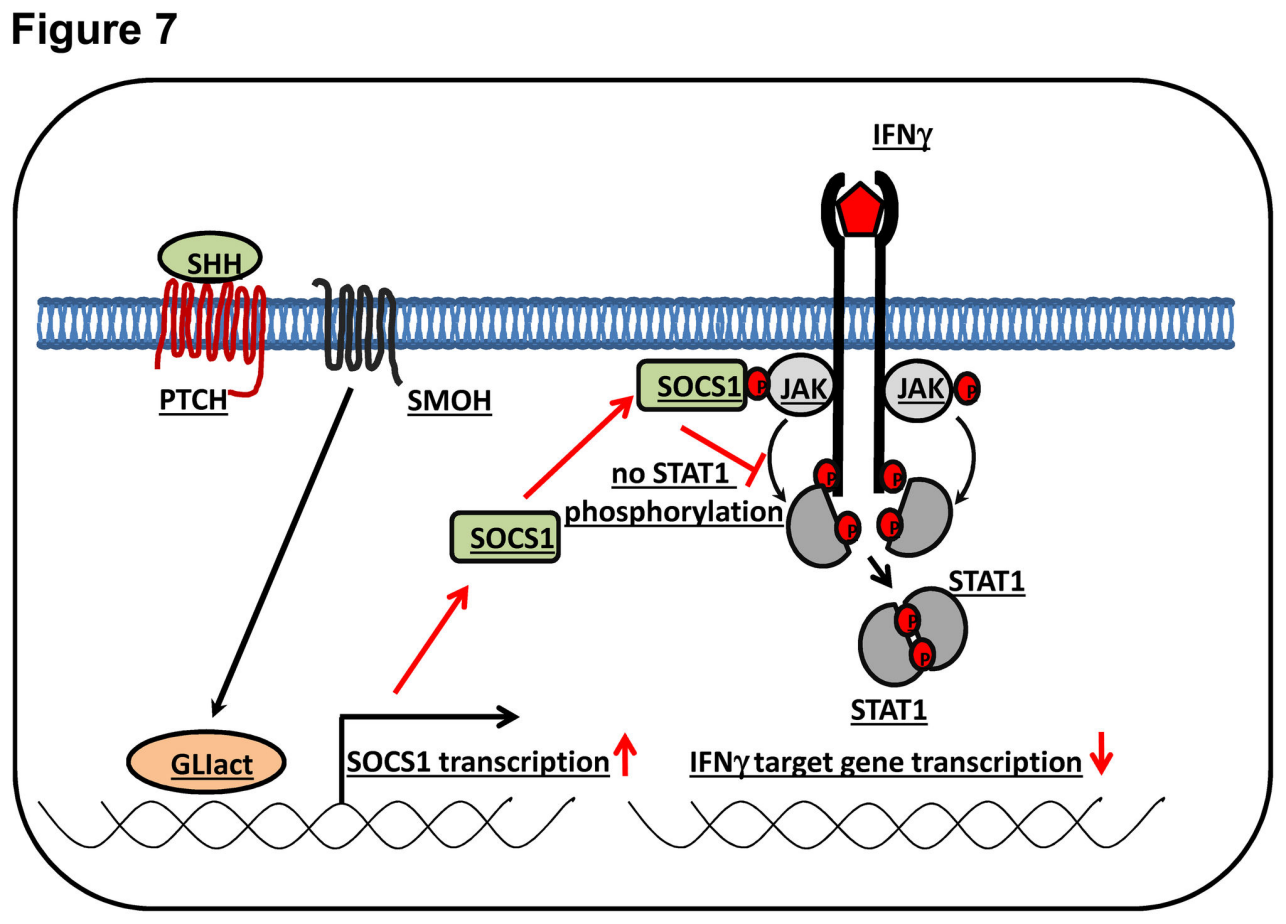
Model showing negative cross talk of Hh signaling with the IFN-у /STAT1 signaling cascade. Activation of Hh/GLI signaling enhances SOCS1 transcription, thereby downregulating IFN-у signal transduction by circumventing STAT1 phosphorylation and dimerization.

In summary, we found that Hh signaling mediated by GLI directly upregulates SOCS1 expression, leading to inhibition of IFN-y signaling (Figure 7). These results may contribute to the understanding how Hh dependent tumors evade cellular anti-tumor strategies relying on IFN-y.

## Materials and Methods

### Ethics statement

The study was performed according to the Austrian Gene Technology Act and in accordance with the Helsinki Declaration of 1975 (revised 1983) and the guidelines of the Salzburg State Ethics Research Committee, being neither a clinical drug trial nor an epidemiological investigation. Concerning this study, the local ethic committee (Salzburg State Ethics Research Committee) was conducted. An objection was not expressed and a waiver of approval was issued (according to the hospital law of Salzburg (§30)). All patients signed an informed consent concerning the surgical removal, therapy of the tumors and use of the residual material within the tissue bank of Salzburg (research purpose). Furthermore, the study did not extend to examination of individual case records. The anonymity of the patients’ samples has been ensured.

### Cloning of promoter and expression constructs

For the luciferase reporter construct SOCS1prom, a 1822bp fragment of human SOCS1 (NM_ 003745.1) promoter was amplified by PCR (forward primer: 5´ gagggtaccggtctccttgtattccatcaccctc 3´, reverse primer 5´ gagagatctccgactcctggctgccctggactcc 3´) from human genomic DNA, digested with KpnI/BglII and cloned into the luciferase reporter vector pGL3basic (Promega). To delete potential GLI binding sites, SOCS1prom was digested with KpnI/SacI and re-ligated resulting in the deletion construct SOCS1promdel. GLI1 and GLI2act expression constructs have been described previously [70].

For the expression plasmid pLL-SOCS1, the open reading frame of human SOCS1 was amplified from human cDNA using PCR (forward primer: 5’ gagaagcttgtagcacacaaccaggtgg 3’, reverse primer: 5’ ctcgaattctcaaatctggaaggggaag 3’), digested with HindIII/EcoRI and sub cloned into pCMV10-3xFLAG (Sigma) to fuse a FLAG tag to the N-terminus of SOCS1. FLAG-SOCS1 was again amplified by PCR (forward primer: 5’ agtcaccggtgtgggaggtctatataag 3’, reverse primer: 5’ gcatgaattctcaaatctggaaggggag 3’), digested with AgeI/EcoRI and cloned into the retroviral expression vector pLL3.7 (pLL) [71].

### Cell culture and colony formation assays

HaCaT and GLI1- and GLI2act-HaCaT [42,72] cells were cultured in Dulbecco’s modified Eagle medium (DMEM, high glucose, PAA) with 10% fetal calf serum (PAA), 100 µg/ml streptomycin and 62.5 µg/ml penicillin (Invitrogen) at 37°C, 5% CO_2_. DAOY cells were cultured in MEM (PAA) with 10% fetal calf serum (PAA), 110mg/l Na-Pyruvate, 100 µg/ml streptomycin and 62.5 µg/ml penicillin (Invitrogen) at 37°C, 5% CO_2_. Transgene expression in GLI1/2act-HaCaT cell lines was induced adding 50ng/ml doxycycline (DOX) (Sigma-Aldrich). During transgene expression or treatment with smoothened agonist SAG (400nM) (Calbiochem) or human recombinant IFN-у (1ng/ml Medium) (Thermo Scientific) serum concentration was reduced to 0.5%. N/TERT-1 cells were grown in Keratinocyte Serum-free Medium (Invitrogen) with 100 µg/ml streptomycin and 62.5 µg/ml penicillin, For the GLI2act-inducible DAOY cell line GLI2act-DAOY, the T-REX System (Invitrogen) was used, generating a double-stable cell line expressing human GLI2act. DAOY cells used already contained the Tet-repressor plasmid and were a kind gift of Dr. Marcel Kool [73]. Medium of double-stable DAOY was supplemented with 10 µg/ml mg/ml Zeocin (Invitrogen) and 2 µg/ml/ml Blasticidin S (Sigma-Aldrich). Transgene expression was induced by 100ng/ml doxycycline (Sigma-Aldrich).

In order to analyze anchorage-independent growth, 8x10^3^ cells were seeded into 12-well plates in 0.4% select agar on top of 0.5% bottom select agar (Invitrogen) according to manufacturer’s protocol. Cultures were grown for 6 weeks at 37°C, 5% CO_2_. Documentation of anchorage independent growth was done using a stereomicroscope (Optimax) and the Cell^D Image capture system (Olympus), for quantification CellProfiler software (http://www.cellprofiler.org/) was used.

### Retroviral gene expression and short hairpin RNA (shRNA) mediated knock down

Lentiviral vectors used for expression of EGFP (pLL) [71] and EGFP tagged GLI1 and GLI2act (pLL-GLI1, pLL-GLI2act) are described in [74]. For shRNA mediated SOCS1 knock down, two shRNAs (shSOCS1_1 (TRCN0000057063), shSOCS1_2 (TRCN0000057067)) from the lentiviral MISSION® shRNA set SHGLY-N2M_003745 (Sigma-Aldrich) and a non-target control shRNA (shCTRL) (Sigma-Aldrich) were selected. Virus production and infection of cells was performed essentially as described in [42]. 24 h post virus infection medium was supplemented with 1 µg/ml puromycin (Sigma Aldrich) to select for infected cells.

### RNA isolation and qRT-PCR analysis

Total RNA from human BCC (n = 7) and human normal skin (n = 3) was isolated with TRI-Reagent (Molecular Research Center, Inc.) followed by LiCl precipitation. Total RNA of HaCaT and N/TERT-1 cells was isolated and purified with the High Pure RNA Isolation Kit (Roche). cDNA synthesis and qRT-PCR analysis was done as described [42]. Human large ribosomal protein P0 (RPLP0) was used for normalization of sample material in qRT-PCR analysis [75]. For primer sequences see Table 1.

**Table 1:** qRT-PCR and ChIP primer sequences.

**Gene name**	**forward (5’ -3’**)	**reverse (5’ -3’**)
**GLI1**	[45]	[45]
**PTCH**	[45]	[45]
**RPLP0**	[75]	[75]
**IFIT1**	AGGCCTTGCAGGAAACACCCACTTC	TTCTGCCCTCTAGGCTGCCCTTTTG
**ICAM1**	ACCCCCATGAAACCGAACACACAAG	CACCAATATGGGAAGGCCGAGGAAG
**TRIM22**	CCCACAGGAGGGGCAGAAGAGAGAT	GCGGAATGTTTGGTGACCTTGGTGT
**SOCS1**	GGCTGGCCCCTTCTGTAGGATGGTA	GGAGGAGGAAGAGGAGGAAGGTTCTGG
**CDKN1A**	CTGGAAGGGGAAGGGACACACAAGA	AGGAAGGTCGCTGGACGATTTGAGG
**CXCL10**	GGCTGCCTCTCCCATCACTTCCCTA	GCAGCTGATTTGGTGACCATCATTGG
**HLA-DRA**	GACTGTGGGTCTGGTGGGCATCATT	CCTGCGTTCTGCTGCATTGCTTTT
**IRF1**	CGCATGAGACCCTGGCTAGAGATGC	CCTTGTTGATGTCCCAGCCATGCT
**IFN-y**	CAGGGGCCAACTAGGCAGCCAAC	TGGAAGCACCAGGCATGAAATCTCC
**RPLP0prom**	TTTTAGTTTGCTGAGCTCGCCAGGTG	CGCCTTTAAGTAGGGTCGCGAAGGA
**PTCHprom**	GAGGATGCACACACTGGGTTGCCTA	GGGCTGTCAGATGGCTTGGGTTTCT
**SOCS1prom F1**	GGGCTCCTCACATGCCTTCATTCAA	TCAAAGACTGCCTTCCCACCACACA
**SOCS1prom F2**	TGCACCTCTCCCCAAAATGAAGACG	ACTACCCGGCTGCGGAAGAAACTGA

### Western blot and Immunohistochemistry

Cells were lysed in 125mM Tris (pH 6.8), 5% glycerol, 2% SDS, 1% β-mercaptoethanol, 0.006% bromphenol blue, and proteins resolved by SDS-PAGE. EGFP, GLI2, SOCS1, total STAT1, STAT1Tyr7001 and ACTB were detected using the following primary antibodies: polyclonal rabbit-anti-GLI2 (GLI2-H300), polyclonal rabbit STAT1p84/p91 (E23) and monoclonal mouse ACTB (C4) all from Santa Cruz Biotechnology, polyclonal rabbit anti SOCS1 (ZymedLaboratories) and p-polyclonal rabbit STAT1 (Y701) (Cell signaling). Secondary antibodies were HRP-conjugated goat-anti-rabbit, chicken-anti-goat (Santa Cruz Biotechnology), and sheep-anti-mouse (GE Healthcare). Proteins were visualized using the SuperSignal West detection system (PIERCE). Paraffin embedded sections of human BCC were stained for SOCS1 as described [70] using a polyclonal rabbit SOCS1 (C20) antibody (Santa Cruz Biotechnology).

### Luciferase Reporter Gene Assay

HaCaT cells were grown in 24-well plates to 80% confluency, and transfected in triplicate with the respective expression constructs and pGL3 basic luciferase reporter plasmids. A lacZ expression plasmid was co-transfected for normalization. Transfection was carried out using SuperFect transfection reagent (Qiagen) according to manufacturer’s protocol. Luciferase activity in cell lysates was measured 48h after transfection with a Lucy IIluminometer (AnthosLabtec) using Luciferase Assay Substrate (Promega) according to manufacturer’s instructions.

### Chromatin immunoprecipitation

Chromatin immunoprecipitation from GLI2-HaCaT cells was done as described in [70]. For immunoprecipitation polyclonal goat-anti-GLI2 (N-20) antibody and normal goat IgG (both Santa Cruz Biotechnology) were used. Sequences of PCR primers used for analysis are listed in Table 1.

### Statistical analysis

Data are shown as mean ± SD. The significance of mean comparison was assessed by two tailed Student’s t test. If not indicated otherwise, the p-value was less 0.05.

## Supporting Information

Figure S1 **DAOY cells express endogenous IFN-y.** RT-PCR measurement showed that untreated DAOY cells express low, but detectable levels of endogenous IFN-y. The house keeping gene RPLP0 was used as reference. Data are given as mean ± SD of biological duplicates.
(TIF)
