# Supplementary figures and images for: Hedgehog/GLI Signaling Activates Suppressor of Cytokine Signaling 1 (SOCS1) in Epidermal and Neural Tumor Cells
Source: PLoS One. 2013 Sep 10;8(9):e75317. doi: 10.1371/journal.pone.0075317 (PMC3769249; doi:10.1371/journal.pone.0075317)

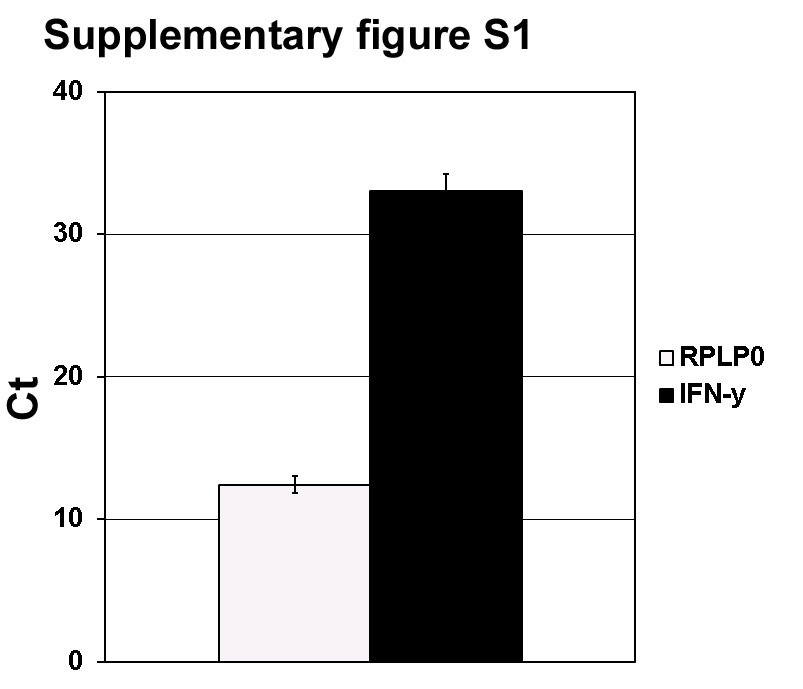

Supplement: Figure S1 — DAOY cells express endogenous IFN-y. RT-PCR measurement showed that untreated DAOY cells express low, but detectable levels of endogenous IFN-y. The house keeping gene RPLP0 was used as reference. Data are given as mean ± SD of biological duplicates. (TIF) [file pone.0075317.s001.tif]
